# The impact of intensive care strain on patients’ outcomes—a multinational observational cohort (UNITE-COVID) study

**DOI:** 10.1186/s13054-025-05521-5

**Published:** 2025-07-28

**Authors:** Katharina Kohler, Thomas De Corte, Massimiliano Greco, Pedro Povoa, Maurizio Cecconi, Marlies Ostermann, Jan De Waele, Andrew Conway Morris, the UNITE-COVID investigators

**Affiliations:** 1 Perioperative, Acute, Critical Care and Emergency Medicine section, Department of Medicine, University of Cambridge, Level 4 Addenbrooke’s Hospital Hills Road, Cambridge, UK; 2 Department of Anaesthesia, Addenbrooke’s Hospital, Cambridge, UK; 3 Dept of Internal Medicine and Pediatrics, Faculty of Medicine and Health Sciences, Ghent University, Ghent, Belgium; 4 Department of Hematology, Ghent University Hospital, Ghent, Belgium; 5 Department of Biomedical Sciences, Humanitas University, Via Rita Levi Montalcini 4, Pieve Emanuele, Milan, 20072 Italy; 6 IRCCS Humanitas Research Hospital, Via Manzoni 56, Rozzano, Milan, 20089 Italy; 7 NOVA Medical School, CHRC, NOVA University of Lisbon, Lisbon, Portugal; 8 Department of Intensive care, Hospital de São Francisco Xavier, CHLO, Lisbon, Portugal; 9 Research Unit of Clinical Epidemiology, Department of Clinical Research, OUH Odense University Hospital, Odense, Denmark; 10 Department of Intensive Care, Guy’s & St Thomas’ Hospital, London, UK; 11 Department of Intensive Care Medicine, Ghent University Hospital, Ghent, Belgium; 12 JVF Intensive Care Unit, Addenbrooke’s Hospital, Cambridge, UK

**Keywords:** Intensive care, Health services, System strain, Healthcare providers, COVID-19

## Abstract

**Purpose:**

Intensive care unit (ICU) strain is associated with increased mortality. Most strain metrics focus on ‘simple’ measures such as bed occupancy or admission rates. There is limited data on mitigation strategies, such as procedure teams or staff well-being services on strain, or the impact of increased patient-to-nurse ratios and non-ICU trained nurses working in ICU.

**Methods:**

Using the multi-national UNITE-COVID study, collecting data from ICUs on their day of peak bed occupancy in two periods (2020 and 2021) of the COVID-19 pandemic, we evaluated metrics of strain (Bed occupancy, patient: nurse ratio, use of non-ICU staff and shortages of consumables) and potential mitigators (procedural support teams and staff well-being interventions). We examined how these related to outcomes (mortality, complications, length of stay).

**Results:**

In both epochs, ICUs experienced significant strain, with ICU bed expansion to 133% and 163% respectively, whilst patient-to-nurse ratios increased by 0.4 and 0.3. Consumable shortages were widespread in 2020. Mortality was inversely correlated with staff well-being interventions in both epochs. Complications were inversely correlated with procedure support teams, and positively correlated with staffing ratios. In regression models, pressure sores were reduced in presence of support teams (*p* = 0.004) and increased with increasing patients per nurse (*p* = 0.05) whilst unplanned extubations were related to non-ICU trained staff working in ICU(*p* = 0.02).

**Conclusions:**

COVID-19 induced ICU strain had effects beyond mortality, including increases in complications. Staff pressure and lack of ICU training were related to specific complications, whilst support teams and well-being interventions were associated with improved outcomes.

**Supplementary Information:**

The online version contains supplementary material available at10.1186/s13054-025-05521-5.

## Background

Strained health systems are those that cannot meet the World Health Organisation (WHO) requirements for healthcare to be high-quality and person centered, timely, equitable, integrated, and efficient [1]. Previous studies investigating the links between health system strain, in particular intensive care unit (ICU) strain, and patient outcomes have found that increased strain is associated with worse patient outcomes, prolonged admissions and increased risk of complications [2, 5]. Most commonly used strain metrics focus on readily measurable elements such as an increase in ICU occupancy or an increase in the proportion of new admissions [6, 9]. The “activity index” integrates a number of these measures, but remains a fundamentally “process” focused measure [10, 11].

The COVID-19 pandemic resulted in exceptional health system-wide stress on resources, particularly with respect to intensive care. In contrast to more ‘routine’ service strain, it forced ICUs to increase patient: staff ratios, use non-ICU trained staff and work in improvised ‘surge’ units. These demands were particularly acute throughout the “first wave“ in winter and spring of 2020 and then again during the “second wave” in winter of 2021. Studies investigating the specific effects of COVID strain on ICU care and outcomes have found some correlation between strain and adverse outcomes [12]. While these studies provide relevant information on this important topic, these again focused on ‘simple’ strain metrics such as bed census and admission rates. Additionally, study outcomes were frequently limited to length of stay and mortality [13, 14] and restricted to either a single center or single country [15].

The UNITE-COVID study was a multi-centre, international point prevalence study examining the burden of COVID-19 in ICUs around the world during 2020 and 2021. By combining centre-level data with the granular patient data, the UNITE-COVID project presents a unique opportunity to comprehensively evaluate strain during the aforementioned COVID-19 periods on ICUs worldwide.

## Methods

### Data collection

The UNITE-COVID data collection and data from 2020 to 2021 editions have been described previously [16]. Sites collected data regarding patients in ICU with COVID-19 on their day of peak occupancy in 2020 (window for peak day identification from February 15th to May 15th) and again in 2021 (window January 1 st to June 1 st) via an electronic case report form (CRF), which can be found in the Online Supplement (Figure S1). Data included patient-level data covering demographic features, comorbidities, severity of illness and complications. Center level data covered hospital and ICU type, changes in ICU capacity and staffing, availability and shortages of resources, visiting policies and modes of communication with family members, availability of task-specific teams and provision of well-being services. Strain measures were selected by the steering committee on the basis of existing literature and clinical experience during the early stages of the pandemic.

### Severity scores and outcome metrics

As described in previous papers, scores were developed to understand the severity of COVID-19 [17, 18] and the burden of comorbidities in the UNITE-COVID cohorts. For comorbidity scoring, each condition was given a weight of 1, within the patient population to enable comparison between different settings. Severity of COVID-19 was determined by ‘ventilation severity score’ calculated based on the level of respiratory intervention needed for each patient: non-invasive ventilation (1), invasive ventilation (2), need for neuro-muscular blockade and/or extracorporeal membrane oxygenation (ECMO) support (3).

To evaluate the effect of shortages (sedative and analgesia medications, ventilation equipment, invasive line insertion provisions, renal replacement therapy, antimicrobials, tracheostomy equipment and “other” shortages), an overall shortage score was calculated by adding each category with equal weight, with shortages resulting in change in practice reported separately from those that did not.

The outcome measures for this analysis were unit average length of stay in ICU, mortality rate and the frequency of potentially avoidable complications, the latter used as a metric of quality of care. The preventable complications were: accidental extubations, thrombotic events, pneumothorax, endotracheal/tracheostomy tube obstructions and pressure sores. These were aggregated with equal weight into a ‘complication score’.

### Strain parameter development

In the absence of a well-established metric of ICU strain we utilized the parameters collected at ICU and individual patient level to develop individual and composite measures across several domains to estimate comparative ICU strain across the cohort and time periods examined. The details of these are set out in the supplemental methods.

### Statistical analysis

All statistical analyses were performed using R studio (v 4.2.1) [19]. Continuous variables are presented as mean and standard deviation for normally distributed data and as median and interquartile range for non-normally distributed data. Categorical variables are presented as percentages and number of evaluable instances (between brackets) unless explicitly stated otherwise. Chi-squared test, Wilcoxon rank sum test and Spearman’s Rho were used where appropriate. A p-value < 0.05 was deemed statistically significant. In case of multiple testing, a Benjamini-Hochberg correction was performed. Variable selection for regression was performed via Lasso regression and implemented as a linear model.

### Ethical approvals

The study received approval from Ghent University Hospital Ethics committee, registration BC-07826 and appropriate approvals at each participating site in line with local regulations (ClinicalTrials.gov registration: NCT04836065, retrospectively registered April 8th 2021).

## Results

### Patient level data

Overall, in 2020, we had data from 4976 patients across 280 ICUs, spanning 45 countries and 5 continents. In 2021, participation was lower, but remained substantial and broad-based with 2503 patients from 37 countries across 5 continents. 69 ICUs participated in both years, providing a comparison for “matched units”.

Patient-level data is presented in Table 1. Despite the changes in provision of evidence-based therapeutics [18], overall mortality was higher in 2021. There was considerable variation in ICU mortality between units and years with median 26.3% [18.2–46.2] in 2020 vs. 28.1% [21.2–44.4] in 2021 (supplemental Figure S2A), as well as in length of stay with median 19.5 [15.5–24.1] days in 2020 vs. 17.8 [13.6–23.3] days in 2021 (Figure S2B).

**Table 1:** Patient level data 2020 and 2021

	2020	2021	*p*-value*	q– value^$^
	N = 4,976	N = 2,503		
*Demographics*				
Sex	71% (3519) Male − 29% (1438) Female(4,957)	67% (1657) Male– 33% (816) Female (2,473)	< 0.001	< 0.001
Pregnancy	0.7% (33/4735)	1.3% (31/2415)	< 0.001	< 0.001
Median age (years)	62 [53–70] (4,891)	62 [53–71] (2,473)	0.055	0.079
Median BMI	28.0 [25.3–32.3] (4,516)	28.1 [25.4–32.3] (2,342)	0.7	0.7
Health care worker	5.6% (255/4,554)	2.6% (55/2,131)	< 0.001	< 0.001
Admission to surge capacity bed	43% (1973/4,589)	59% (1461/2,476)	< 0.001	< 0.001
*Comorbidities*				
Chronic cardiac disease	16% (760/4,751)	17% (411/2,417)	0.11	0.15
History of hypertension	50% (2383/4,766)	52% (999/2,423)	0.030	0.048
Chronic liver disease	2.6% (123/4,739)	3.9% (94/2,423)	0.004	0.008
Chronic neurological disease	5.9% (279/4,737)	7.1% (172/2,421)	0.063	0.087
Chronic pulmonary disease	9.0% (428/4,752)	12% (290/2,413)	< 0.001	< 0.001
Diabetes			0.4	0.5
* No diabetes*	68.4% (3246/4,746)	68.8% (1657/2,408)		
* Type I diabetes*	2.1% (100/4,746)	1.6% (38/2,408)		
* Type II diabetes*	29.5% (1400/4,746)	29.6% (713/2,408)		
Asthma	8.8% (419/4,761)	8.7% (211/2,425)	0.9	0.9
Malignant neoplasm	5.5% (259/4,700)	7.0% (169/2,410)	0.016	0.027
Chronic kidney disease	7.1% (338/4,756)	8.8% (21.3/2,423)	0.012	0.022
Immunosuppression	5.1% (240/4,704)	6.6% (159/2,412)	0.008	0.015
HIV			0.083	0.11
* HIV– not on ART*	0.3% (13/4,409)	0.7% (16/2,289)		
* HIV– on ART*	0.4% (18/4,409)	0.3% (7/2,289)		
* No HIV*	99.3% (4378/4,409)	99% (2266/2,289)		
Comorbidity score^&^	(4409)	(2289)		
0	33% (1455)	30% (687)		
1	29% (1279)	30% (689)		
2	20% (882)	19% (435)		
3	8.4% (370)	10% (229)		
4	6.2% (273)	6.8% (156)		
5	2.6% (115)	2.8% (64)		
6	0.7% (30)	1% (24)		
7	< 0.1% (5)	0.1% (5)		
*Chronic medication*				
ACE-inhibitor	19% (862/4,538)	19% (433/2,281)	0.6	0.7
Angiotensin II receptor antagonist	15% (679/4,529)	17% (387/2,276)	0.045	0.069
Anticoagulation	6.9% (316/4,580)	11% (253/2,299)	< 0.001	< 0.001
Antiplatelet therapy	17% (776/4,567)	17% (253/2,301)	0.5	0.6
*Clinical status at ICU admission*				
Reason for admission			< 0.001	0.002
* Referral from another ICU*	7.7% (370/4,807)	6.2% (153/2,468)		
* ICU admission due to respiratory failure*	88% (4230/4,807)	88% (2167/2,468)		
* ICU admission due to other complications of COVID-19*	2.2% (106/4,807)	3.3% (81/2,468)		
* ICU admission due to other diagnosis*	2.1% (101/4,807)	2.7% (67/2,468)		
Median [IQR] days between symptoms and hospital admission	7.0 [4.0–9.0] (4,234)	6.0 [4.0–9.0] (2,065)	< 0.001	< 0.001
Median LoS in hospital before ICU admission	1.0 [0.0–4.0] (4,676)	2.0 [0.0–4.0] (2,302)	0.003	0.007
Respiratory support before ICU admission (any)	73% (3509/4,807)	76% (1853/2,438)	< 0.001	0.002
*Type of pre-ICU respiratory support*				
* CPAP*	11% (354/3,220)	15% (256/1,707)		
* HFNO*	7.8% (251/3,220)	21% (358/1,707)		
* NIV*	5.2% (167/3,220)	11% (188/1,707)		
* Standard oxygen*	76% (2447/3,220)	53% (905/1,707)		
Median days of respiratory support before ICU admission	1.00 [1.00–3.00] (709)	2.00 [1.00–3.00] (773)	< 0.001	0.002
Deep vein thrombosis at admission	0.8% (38/4752)	1.0% (25/2,502)	0.4	0.5
Pulmonary embolism at admission	1.9% (90/4752)	4.9% (123/2,502)	< 0.001	< 0.001
Other thrombosis at admission	1.4% (66/4752)	2.7% (70/2,502)	< 0.001	< 0.001

Medians are reported with the interquartile range IQR in []

*ART * Anti-retroviral treatment, *CPAP * Continuous positive airway pressure, *HFNO* High flow nasal oxygen, *NIV* Non-invasive ventilation

* Chi-squared test or Wilcoxon rank sum test was used where appropriate

^$^ q-value was determined using the false discovery rate (Benjamini-Hochberg) procedure for multiple testing

^&^ Comorbidity score achieved by summing the number of co-morbidities recorded (chronic cardiac disease, arterial hypertension, chronic pulmonary disease (excluding asthma), asthma, chronic liver disease, chronic kidney disease, diabetes mellitus, malignancy, immunosuppression)

In 2020, 217 centres had sufficient data for analysis, whilst 2021 it was 87 (Supplemental Figure S3 shows inclusion flowchart) 21 units had sufficient data for all outcomes in 2020 and 2021 (see, for instance, Table S1). In 2020, centers included between 1 and 121 patients into the study with a median of 13 [7–23], and in 2021, there were a median of 18 patients [10.5–25]. Aggregating overall patients constitutes a weighting towards the larger centers, however when aggregated at centre level (therefore weighting each centre equally) there were few major differences.

The severity of illness was summarized by the ventilation severity score [17, 18]. When aggregated by centre, in 2020 the mean ventilation severity score was 2.2 +/- 0.6, reflecting the severity of illness of the population and the significant contribution of ECMO centres to the study. The mean comorbidity score was 1.0 +/- 0.6 showing the level of background comorbidities to be relatively low in the patient population. In 2021, similarly the mean ventilation score was 2.3 +/- 0.6 and the comorbidity score was 1.0 +/- 0.5.

### Centre data

Supplemental Table S1 shows the number of centres, number of patients and the bed state characteristics during the 2020 and 2021 surge periods.

Whilst hospitals and ICUs expanded bed capacity in both years, the surge in ICU beds in 2020 was smaller than overall hospital capacity increase (33% vs. 56% increase over baseline respectively). In 2021 this was inverted as the ICU bed capacity surged by 63% while the overall hospital beds increased by 53%, with similar findings when only comparing matched units (Table S1), potentially showing that centres were better equipped and prepared for the needed ICU capacity. Additional features describing the composition of units in terms of provider type and open vs. closed were broadly similar between 2020 and 2021 (see supplemental Figure S4).

There was an increase patient-to-nurse ratio (rising from baseline median 1.9:1 to surge 2.3:1 in 2020 (*p* < 0.001) with similar values in 2021 (*p* = 0.03) (Table 2) and an increase in patient: intensivist ratio that was significant in 2021. Additionally, a significant number of centres deployed non-ICU trained nursing staff into ICUs. The matched centres data is shown for validation, of note is the slightly changed baseline of the patient: nurse ratio standard between 2020 and 2021, however the IQRs overlap and the data was collected as a snapshot with potential variations depending on day/week.

**Table 2:** Staffing related factors 2020 and 2021

	2020– all centres (204)	2021– all centres (94)	2020– matched centres only	2021– matched centres only
Patient: nurse ratio standard	1.9 [1–2]	1.8 [1–2]	2.4 [2–3]*	1.8 [1.5–2]*
Patient : nurse ratio surge	2.3 [2–3]	2.1 [2–2.5]	2.9 [2–4]	2.0 [2–2]
Patient: intensivist ratio standard	3.6 [3–9]	3.6 [2.8–9]	4.9 [4–10]	3.2 [3–9.25]
Patient: intensivist ratio surge	4.2 [3.9–10]	5.2 [4–12]	5.9 [5–9]	5.2 [4–12]
Percentage of centres with non-ICU trained nurses working in ICU	74% (151)	71% (67)	59% (12)	67% (14)

* Baseline staffing changed between 2020 and 2021

**Fig. 1:**
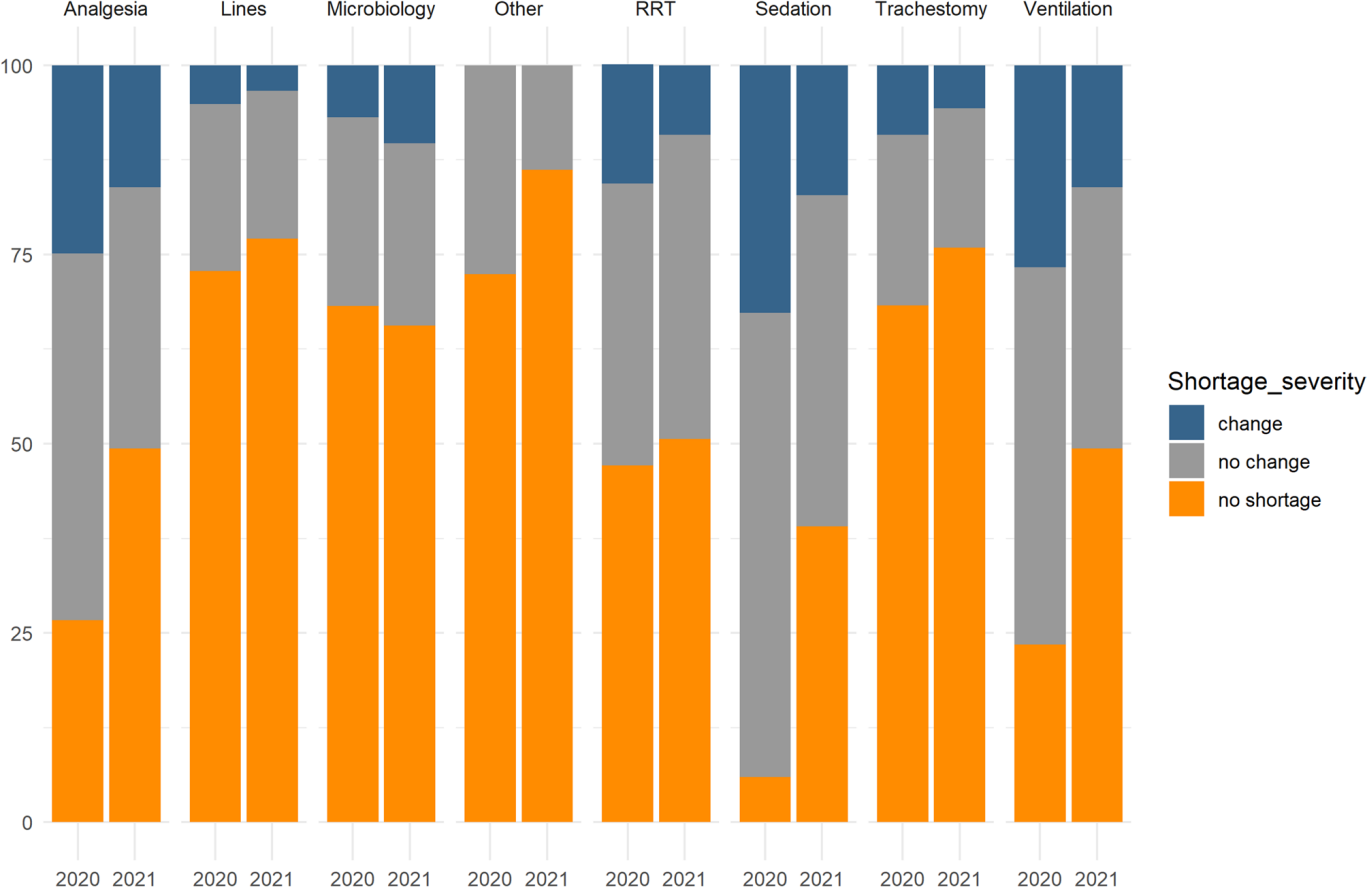
Shortages stacked bar chart: Showing the shortages across both surge periods in all recorded categories. RRT = renal replacement therapy. Change = shortages that caused a change in practice, No change = shortages that existed but did not cause a change in practice. Notable is that shortages with change decreased in 2021 compared to 2020. Sedation/analgesia medications and the ventilation/renal replacement treatments were most affected by shortages

Information on resource shortages showed widespread shortages of medications and equipment in 2020, although the majority did not impact on practice (Fig. 1, Table S2). The surge in 2021 again saw shortages, although to a lesser degree than in 2020. The main shortages with an impact on practice occurred with ventilators and sedation and analgesic medications.

Analysis of the overall average shortage score with a change in practice showed a median of 0 [0–4] for 2020 and 0 [0–1] for 2021. The higher shortages leading to a change in practice in 2020 represented a significant difference to the lower ones in 2021 (*p* = 0.02).

**Fig. 2:**
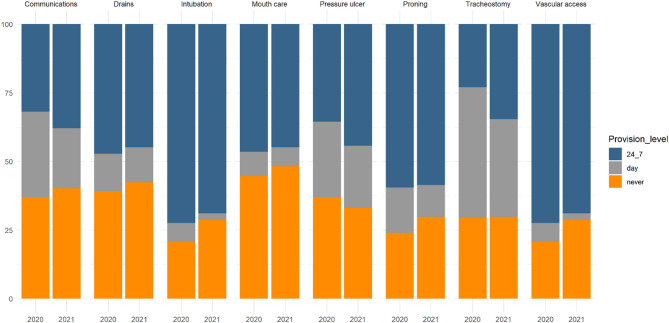
Shows the support team provisions in the two time periods. Overall team support score was 5.5 [IQR 207] in 2020 and 5 [IQR 1.25–7.5] in 2021

2020 saw extensive use of specialist support teams, most notably intubation, vascular access and proning teams (Fig. 2). 2021 saw a modest reduction in such team provision. Table S3 compares the matched units, showing similar trends.

We examined staff welfare provisions (Table S4) that aimed to mitigate strain. 2020 saw high levels of support including provision of free food in 82% and free accommodation in 52% of centres, and psychological support in 68%. In 2021 only the psychological support was maintained at similar levels to 2020, indeed showing a significant increase in matched centers, whilst other forms of welfare support were less frequently offered.

**Fig. 3:**
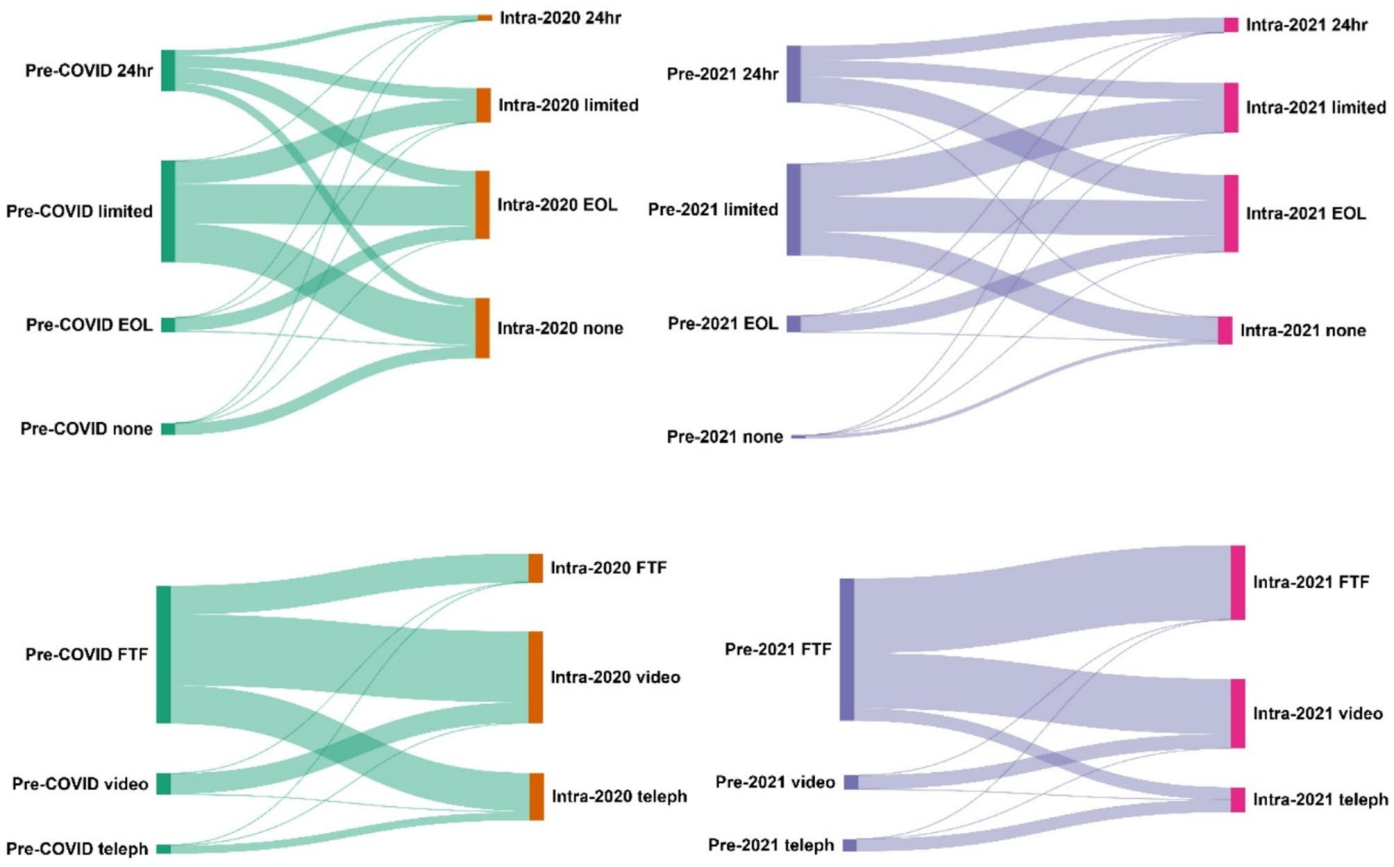
Changes in visiting policies (top) and relative communication policies (bottom): Pre COVID– prior to surge 2020, Intra 2020– during surge 2020, Pre 2021– prior to surge 2021, Intra 2021– during surge 2021 *Visiting: EOL: end of life. Relative communications: FTF– face to face*,* telephone– via voice (phone or other)*,* video– via video tool*

Communication methods and visiting policies also changed substantially during the surges of 2020 and 2021 (Fig. 3). The overall trend was towards more restrictive visiting during COVID peaks, although this was less marked in 2021. Communication was similarly altered with reduction in face-to-face communications, again this was less marked in 2021.

### Correlation of strain parameters with outcomes

**Fig. 4:**
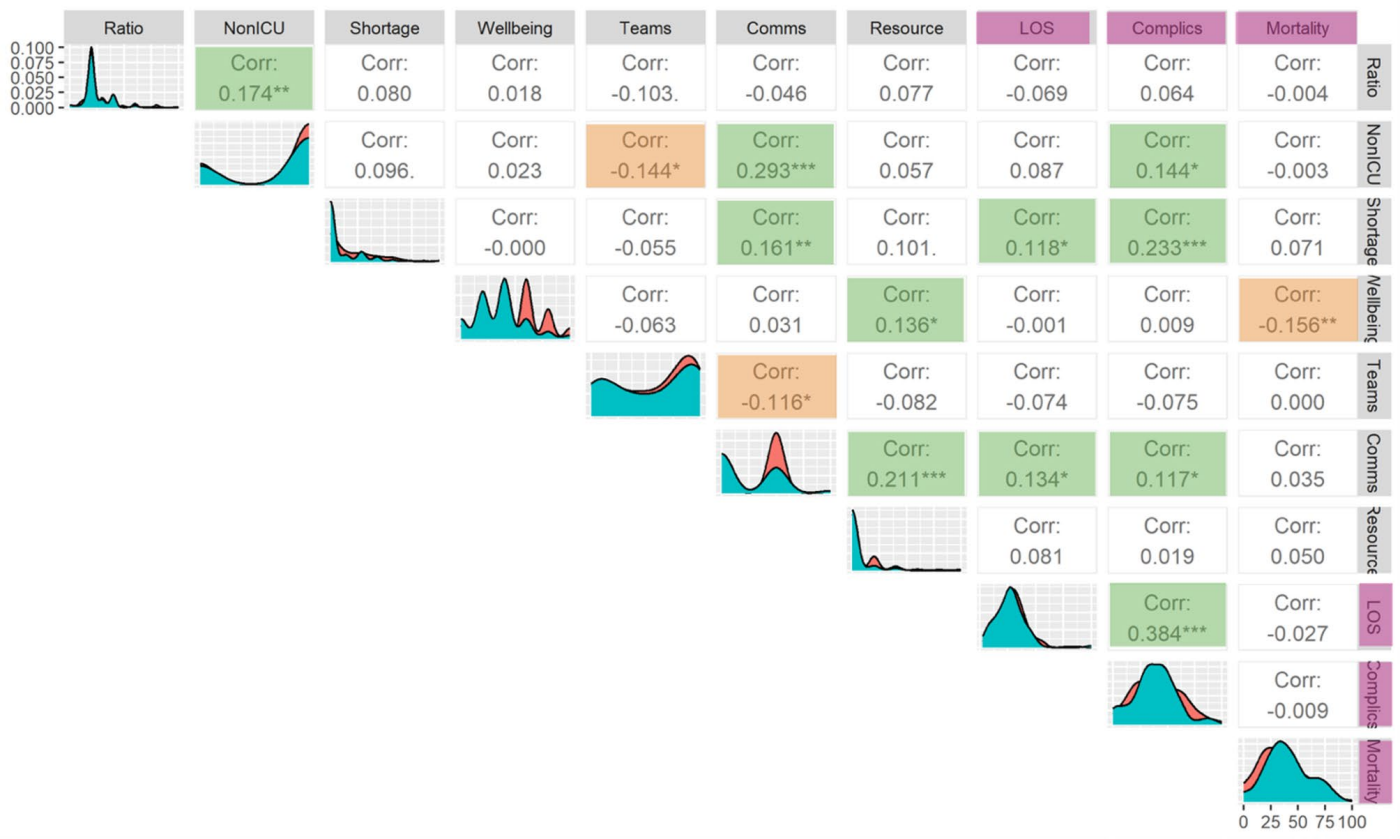
Correlation of outcomes with main strain categories. Correlation matrices show the relation between strain parameters (grey) and outcomes (purple) and relationships within these categories of variable. The diagonal shows the distribution of the variable in question (2020 in red, 2021 in teal), the boxes contain the relevant correlation coefficient for each pair of variables, *p < 0.05, **p < 0.01, ***p < 0.001 by Spearman’s Rho coefficient. Significant positive correlations are highlighted in green, significant negative correlations are highlighted in orange. Ratio– nursing ratio change in surge times (increased ratio indicating more patients per staff), NonICU– non-ICU nursing staff recruited into the ICU, Comms– communication change score, LOS– length of stay, Complics– complication score across domains

To understand the relationships between the strain metrics, and how these relate to our outcomes we show a correlation matrix (Fig. 4, see Figure legend for details) across variables and outcome metrics for both surge periods.

Figure 4 shows that the outcome measures are correlated with a range of aggregated strain scores and staffing metrics, notably non-ICU trained staff working in ICU shortages. Statistically significant correlations also include the negative correlation of well-being support with mortality (*p* < 0.01), indicating a higher mortality in ICUs with lower well-being support.

Complications had a positive correlation with the use of Non-ICU staff. Additionally, the complication score was positively correlated with communication changes. The most significant correlation was with the shortage score [20], indicating that shortages that resulted in a change in practice were associated with higher rate of complications of care. Several strain measures showed significant correlations between themselves, indicating the multi-faceted and interacting nature of strain.

### Regression modelling of strain parameters on outcomes

We sought to further understand the extent to which individual ICU strain metrics explained the variation in outcomes by constructing linear regression models for the outcome variables using the strain variables (Fig. 4) as parameters. To account for differences in patient cohort characteristics we also included the centre mean ventilation severity and comorbidity score. The variables entered into final model were selected using Lasso regression methodology, maintaining only those significantly contributing to the model (see Table S5).

### Complications modelling

The distribution of complications is shown in supplemental Figure S5. For 2020, the linear regression model for the aggregate mean complication score contained the mean ventilation severity score (*p* < 0.001) and the shortage score (*p* = 0.02) with a model R^2^ of 0.19. Both coefficients were positive indicating that higher severity and increased shortage strain related to a higher complication score.

For the 2021 surge period the best fit model contained the mean ventilation severity score (*p* < 0.001) and the income level of the country (*p* = 0.03) with a similar R^2^ = 0.22.

Investigating the sub-categories of complications (Table S5) found that different complications had slightly different best fit models. In 2020 the additional findings were that the model for unplanned extubation complications contained the presence of non-ICU trained staff in the ICU as a significant variable (*p* = 0.04) and the pressure sore complication model included the presence of support teams (*p* = 0.004) and the change in patient to nurse ratio (*p* = 0.05). The presence of support teams reduced the pressure sore score, whereas an increase in patients per nursing staff increased it.

The analysis for 2021 showed similar results with the main additional finding that the mean centre comorbidity score was significant (*p* = 0.05) in the pneumothorax subcategory of complications indicating a higher risk with the presence of increased comorbidities, in addition to the national income level showed significance indicating a relationship between this and outcomes.

### Mortality modelling

The 2020 surge mean centre mortality model included the mean comorbidity score (*p* < 0.001) and the wellbeing support teams (*p* = 0.008) with an R^2^ = 0.10 (Table S5) with the presence of wellbeing teams associated with a lower mortality rate.

The 2021 surge mean centre mortality model included only the mean comorbidity score (*p* < 0.001) with a positive coefficient indicating an increase in mortality with an increase in comorbidities with an R^2^ = 0.12 and no strain parameters showing significant contributions.

### Length of stay modelling

For the 2020 surge period the regression model showed no parameters significant for inclusion. For the 2021 surge period the significant variables in the parsimonious model were the income level of the country (*p* < 0.001) and the change in nursing ratio (*p* = 0.03) with coefficients indicating that length of stay increased in higher income settings and in ICUs with higher patient load per staff.

## Discussion

This secondary analysis of data from the large, multi-continental UNITE-COVID study brings deeper and wider understanding of the effects of the COVID-19 pandemic on ICU working. ICU strain is usually considered within the context of existing bed and staffing envelopes, and in high-income nations, shortages across wide ranges of healthcare consumables are extremely rare. There has also been almost no prior work evaluating mitigation strategies for ICU strain during COVID-19 [21], with pre-pandemic work having been extremely limited and largely focused on very specific areas of practice in single units [22, 23].

Our analysis finds that there were significant changes to service provision throughout the two peak periods examined with a more significant impact of shortages and capacity issues during the first peak (2020). Important changes to working practices occurred during both peaks, some driven by clinical need, such as the widespread practice of adding procedure support teams [24]. Others were driven by shortages of usual medications or equipment [25], whilst infection control concerns motivated the changes in visiting and communication policies [26]. These changes were a pragmatic response to unprecedented pressure, however, there were associations with increased complications when practice had changed due to shortages or with respect to communication policies. Although we did not measure staff mental health in this study, previous studies have found significant levels of psychological distress and moral injury amongst healthcare workers with the most junior staff most strongly affected [27]. Our novel metrics of strain do, however, require further validation in external datasets.

The regression modelling found significant relationships between comorbidity scores and mortality, giving reassurance that the models detect known and plausible relationships. Overall complications were independently associated with shortages and changes in communication strategy, whilst correlation analysis suggested a protective association with procedure teams. Individual complications revealed interesting associations, notably between pressure sores and increased nursing workload and accidental extubation and use of non-ICU trained staff. National income level was also associated with nosocomial infections and length of stay, possibly related differential healthcare resources availability. Whilst we cannot infer causation from these relationships, the divergent relationships do at least suggest that complications were not simply a reflection of strain, and have the potential to be mitigated.

We found that filling staff shortages with non-ICU trained health care professionals was associated with an increased risk of certain complications. This was most prominent in 2020, and it is possible that interventions to improve staff training, such as the ESICM’s C19Space initiative and similar programmes had a positive impact preparing staff better for the subsequent wave in 2021 [28]. Other evidence of better preparedness in 2021 include the relative expansion of ICU beds (Table S1) and reduced shortages and clinically-impactful shortages (Fig. 1 and Table S2). The pressure sore complication rate findings are an example of both risk factors (increase in patients per nursing staff) and mitigation by presence of additional help (support by special teams) indicating potential mitigations to the risks of increased workload and reduced skill mix. The impact of the COVID-19 pandemic on communication and visiting has been reported previously [26], and clinicians reported dissatisfaction with the approaches taken and the potential for detrimental effects on staff, patients and families [26]. Our identification of an association between changed communication and outcomes suggests these concerns were valid.

The role of staff well-being support remains notably under-explored in both the COVID and wider ICU literature. Well-being support across several domains was positively associated with a reduction in mortality. Whilst we cannot infer causality from our data, a recent systematic review of 85 studies found a relationship between nursing burnout and care quality [29]. Attention to staff well-being may help improve patient outcomes both at times of exceptional strain and also during more routine times.

This study has a number of strengths. First it reports results from every continent and from a large number of ICUs. It also identified ICUs at point of maximal bed capacity strain, and therefore is able to identify factors beyond simple bed number and admission rates that impacted on outcomes. Second, due to the unprecedented changes in practice forced on ICUs by COVID-19 we were able to assess the impacts of strain across domains that are seldom examined, namely shortages of medicines and other consumables, radical changes in communication and visiting and widespread recruitment of non-ICU staff to ICU roles.

The limitations of this study include reliance on ICUs self-reporting and we could not externally validate data reported. We were also unable to collect subjective measures of strain experienced by staff. The composition and training of procedure support teams may have varied between sites, and indeed within sites over time, however the relationships seen were robust to such potential heterogeneity. Complications were assessed at site, and although we were unable to ensure standardisation of complication assessment the narrow range of variability (Figure S5A and B) suggests consistent recording of complications. The staff data collection was restricted to professional staff, and did not collect data regarding changes in support staff such as healthcare assistants so cannot determine what relationship, if any, there was between wider staffing changes and outcomes. For some questions, most notably bed number and surge bed number, the question was interpreted in different ways in different ICUs requiring post-hoc adjudication by the investigators as to actual bed number at baseline and expansion. We also must caution against drawing causal inferences from our data, as an observational study, this must be seen as hypothesis generating rather than definitive. We did not differentiate in our analysis between specific ICU types as the analysis was conducted at site level not unit level. It is therefore not possible to distinguish between “surge ICUs” (set up purely during the surge periods) and standard ICUs. Whilst previous studies have shown relationships between ICU strain and outcomes, this work extends this to both less commonly assessed outcomes (complications), demonstrating that these relationships persist across types of ICU, institutions and national boundaries and can be detected even at times of world-wide maximal strain.

## Conclusions

The natural experiment of exceptional ICU demand in 2020 and 2021 found that increased strain, in particular via shortage-incurred changes in practice, were related to worse outcomes. We also found that increasing nurse: patient ratios and deployment of non-ICU nurses to ICUs were associated with increased complications. Improving working conditions for staff via well-being initiatives and procedural support teams provided some mitigation, although whether these relationships are causal requires further investigation. Investigating strain mitigation may help crisis preparedness for ICUs.

## **Take home message**

We examined the effects of various aspects of ICU strain on patient outcomes during the periods of maximal unit occupancy during the COVID-19 pandemic. We identified adverse relationships between preventable complications and increases in patient: nursing ratios and use of non-ICU trained staff, whilst procedural support teams and staff well-being interventions were associated with better patient outcomes.

## **140 character summary**

COVID strained ICUs. Increased patient: staff ratios & non-ICU staff increased complications, staff well-being initiatives improved outcomes.

## Supplementary Information

Supplementary Material 1

Supplementary Material 2

## Data Availability

The datasets used and analysed during the current study are available from the corresponding author on reasonable request. The data is not stored in a publicly available database as it contains potentially identifiable data.
